# Downregulation of POFUT1 Impairs Secondary Myogenic Fusion Through a Reduced NFATc2/IL-4 Signaling Pathway

**DOI:** 10.3390/ijms20184396

**Published:** 2019-09-06

**Authors:** Audrey Der Vartanian, Julien Chabanais, Claire Carrion, Abderrahman Maftah, Agnès Germot

**Affiliations:** 1 PEIRENE, EA 7500, Glycosylation et différenciation cellulaire, Université de Limoges, F-87000 Limoges, France; 2 present address: INSERM, IMRB U955-E10, Faculté de Médecine, Université Paris Est Créteil, F-94000 Créteil, France; 3 UMR CNRS 7276, Contrôle de la Réponse Immune et des Lymphoproliférations, Université de Limoges, F-87000 Limoges, France

**Keywords:** IL-4, myogenic differentiation, NFATc2, NOTCH pathway, POFUT1, secondary fusion

## Abstract

Past work has shown that the protein *O*-fucosyltransferase 1 (POFUT1) is involved in mammal myogenic differentiation program. *Pofut1* knockdown (Po –) in murine C2C12 cells leads to numerous elongated and thin myotubes, suggesting significant defects in secondary fusion. Among the few pathways involved in this process, NFATc2/IL-4 is described as the major one. To unravel the impact of POFUT1 on secondary fusion, we used wild-type (WT) C2C12 and Po – cell lines to follow *Myf6*, *Nfatc2, Il-4* and *Il-4rα* expressions during a 120 h myogenic differentiation time course. Secreted IL-4 was quantified by ELISA. IL-4R*α* expression and its labeling on myogenic cell types were investigated by Western blot and immunofluorescence, respectively. Phenotypic observations of cells treated with IL-4Rα blocking antibody were performed. In Po –, we found a decrease in nuclei number per myotube and a downexpression of *Myf6*. The observed downregulation of *Nfatc2* is correlated to a diminution of secreted IL-4 and to the low level of IL-4Rα for reserve cells. Neutralization of IL-4R*α* on WT C2C12 promotes myonuclear accretion defects, similarly to those identified in Po –. Thus, POFUT1 could be a new controller of myotube growth during myogenesis, especially through NFATc2/IL-4 signaling pathway.

## 1. Introduction

During postnatal skeletal myogenesis, the muscle stem cells called satellite cells play a crucial role in muscle growth, homeostasis and regeneration. Under activation, these cells commit to a myoblast cell fate and allow cell fusion and fiber formation. Initiation of the myogenic program is defined by expression of *Pax3/7* followed by the hierarchized expression of the myogenic regulatory factors (MRFs), *Myf5*, *MyoD*, *MyoG* [1,2] and *Myf6* [3,4], which are necessary for the formation of multinucleated myotubes (MT). For mammals, myoblast (MB) fusion is a critical mechanism in muscle development and regeneration of mature myofibers upon muscle injury in adults [5]. Skeletal muscle fibers arise from two complementary fusion processes of MB [6,7]. Initially, the primary fusion is defined by the alignment of MB, which fuse together leading to the formation of nascent MT. Then, the myonuclear accretion, corresponding to the secondary fusion, results from the recruitment of surrounding MB by the immature MT. Distinct signaling pathways are involved in the myogenic differentiation [8]. Among them, the activation of the NOTCH pathway maintains satellite cells in a quiescent state and contributes to proliferation of MB [9]. The NFATc2/IL-4 (nuclear factor of activated T-cells, cytoplasmic, calcineurin dependent 2/Interleukin-4) pathway is specifically involved in the secondary fusion process [10,11]. It acts on myoblast fusion after the initial formation of MT and is necessary for their growth. Regulated by calcium-dependent signaling, NFATc2 is dephosphorylated and translocates to the nucleus to induce the production of the cytokine IL-4 by nascent MT. The IL-4 receptor alpha (IL-4Rα) expressed at the cell surface of MB allows their recruitment by nascent MT [12]. Furthermore, it is known that *Il-4rα* expression is induced and stabilized in the presence of IL-4 itself [13,14]. *Il-4rα* is overexpressed in severe human diseases such as embryonic and alveolar subtypes of rhabdomyosarcoma (ERMS, ARMS, respectively). In humans, and mouse model, it is involved in tumor metastatic properties [15]. RMSs are common pediatric cancers of soft tissue with a poor prognosis [16]. An aberrant upregulation of NOTCH signaling pathway [17] and Pax3/7 expression [18] are also found in the rhabdomyosarcoma cells to be responsible for the tumor growth. Even if tumors are mostly positive for MYOD and MYOG [19,20,21], which act as skeletal muscle lineage and differentiation markers, RMS cells fail to fuse into mature myofibers [20,21]. As myogenic differentiation and fusion capacities are distinctively, but both impaired, one potential therapeutic strategy could be to combine the IL-4R*α* blockade with an inhibition of NOTCH signaling to target the tumorigenesis of RMS. Recently, we proposed that protein *O*-fucosyltransferase 1 (POFUT1) acts through the NOTCH signaling pathway as a myogenic modulator, which controls the commitment of MB into the myogenic program [22,23]. POFUT1 is an endoplasmic reticulum (ER)-resident enzyme [24] responsible for the *O*-fucosylation of S or T included in EGF-like domains of around eighty secreted or membrane glycoproteins in human or mouse, especially NOTCH receptors and ligands, that contain the consensus sequence C^2^X_4_(S/T) C^3^ (where C^2^ and C^3^ are the second and third cysteines of EGF-like domain, respectively) [25]. *Pofut1*^-/-^ mice die at mid-gestation with severe alterations particularly in somitogenesis, similar to those observed for embryos lacking downstream effectors of the NOTCH signaling pathway [26]. In *Pofut1*^cax/cax^ mice presenting the cax (compact axial skeleton) hypomorphic allele of *Pofut1*, skeletal muscles show a postnatal hypertrophy associated with a decrease in PAX7+ satellite cells [23]. The *O*-fucosylation of NOTCH receptors mediated by POFUT1 is essential for the canonical NOTCH signaling pathway [27]. Abnormal NOTCH signaling has been associated with human POFUT1 dysfunction in various cancers such as breast [28], hepatocellular [29] or colorectal [30] ones where POFUT1 overexpression is correlated with tumor development. Several studies demonstrated in osteoblasts [31] and keratinocytes [32] the interdependence of NOTCH and NFATc2 signaling pathways. The cleavage of NOTCH intracellular domain (NICD) is involved in the stabilization of *NFATc2* transcript via a post-transcriptional mechanism [31]. Moreover, *NFATc2* overexpression suppress canonical NOTCH transactivation and expression of *HEY* (Hairy/Enhancer of Split-related with YRPW motif) genes.

Our study identifies a new critical role of POFUT1 in the secondary fusion process. Downregulation of *Pofut1* in differentiating C2C12 cells induces a myonuclear accretion defect in MT. It results from a decrease of *Nfatc2* mRNA expression, which is associated with a diminution of secreted IL-4 in the culture medium and a reduction of IL-4R*α* quantity for cells normally contributing to MT growth.

## 2. Results

### 2.1. Secondary Fusion Defect Occurs between 72 h and 120 h of Differentiation in Pofut1 Knockdown C2C12 Cells

**Figure 1:**
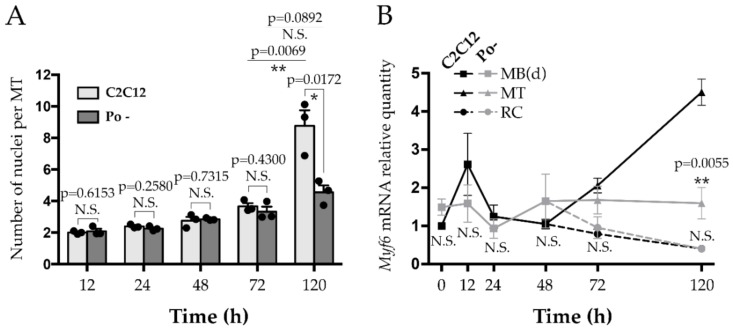
Timing of secondary fusion in C2C12 cell lines. (**A**) Mean number of nuclei in myotubes from wild-type (WT) C2C12 and *Pofut1* knockdown (Po –) cells during myogenic differentiation time course. (**B**) Relative quantities of Myf6 expression in WT C2C12 (black) and Po – cells (grey). During myogenic fusion, some of the differentiating myoblasts (MB(d)) will fuse together leading to the formation of myotubes (MT), while others will generate mononucleated reserve cells (RC) that will participate in MT growth during secondary fusion. All experiments were performed in biological triplicates. In both cell lines, fold changes were relative to time 0 h of WT C2C12. Bar graphs and plots represented means ± SEM (Standard Error of the Mean). In (**A**), each mean of replicates was symbolized by a dark dot. Statistical analyses were done using a two-tailed Student test, which compared within and between differentiation times, and according to both cell types, MT and RC. * *p* < 0.05; ** *p* < 0.01. N.S., not significant.

To evaluate myonuclear accretion process in C2C12 cells, nuclei number was determined during 120 h of differentiation time course in wild-type (WT) and *Pofut1* knockdown (Po –) cell lines; the latter having been created for a previous study [22]. Although no significant difference was observed in the first 72 h of myogenic differentiation between the cell lines, Po – MT had a significant lower number of nuclei at 120 h. They contained around four nuclei per MT, whereas WT MT contained around nine nuclei (*p* < 0.05) (Figure 1A). In the normal differentiation process of C2C12, the number of nuclei in MT was at least multiplied by two between 72 h and 120 h (*p* < 0.01), whereas in Po –, no significant difference was noticed. We already observed [22] that the knockdown of *Pofut1* in C2C12 cell line induced thinner and longer MT than WT ones and a significant increase of MT population containing fewer nuclei compared to WT.

**Figure 2:**
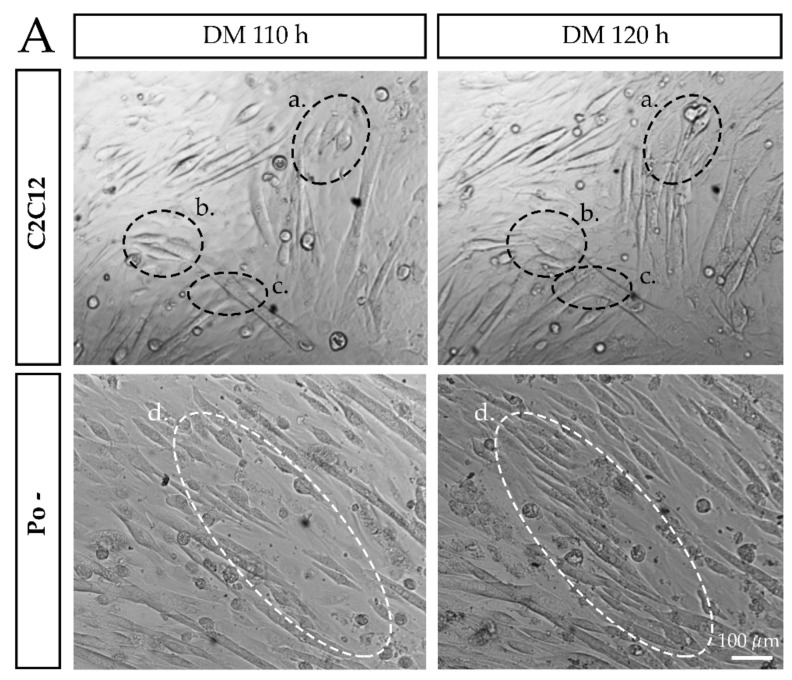
Live-cell monitoring of fusion dynamics in WT C2C12 and Po – cells at 110 h and 120 h of myogenic differentiation. The C2C12 myoblasts, in the process of differentiation, align and fuse together to form nascent myotubes, in the primary fusion step (**a**). Then, some of the surrounding reserve cells fuse with the nascent myotubes participating in their elongation, in the secondary fusion step (**b**). Primary myotubes also fuse together in the secondary fusion process (**c**). In the case of Po – myoblasts, they align and rapidly fuse into elongated myotubes, but only include a small number of nuclei (**d**). Thus, the surrounding reserve cells will not fuse with these atypical myotubes and lengthen them.

The maturation of MT was evaluated by *Myf6* gene expression measurement during the differentiation time course of WT C2C12 and Po – (Figure 1B). During the first 48 h, expression level of *Myf6* was not significantly different in Po – compared to WT cells. At 72 h, when cell types are distinguishable, no significant difference was observed between cell lines regardless the MT and RC considered. At 120 h, WT MT, but not Po – MT, showed a remarkable increase of *Myf6* expression, around 4.5 times more than in the differentiating myoblasts, MB(d), at 0 h (*p* < 0.01). From 96 h of differentiation, the cell fusion was periodically followed every 15 min until 120 h using a time-lapse microscope, in both WT and Po – cell cultures (Figure 2; Supplementary Video S1a,b). Both WT and Po – cell lines presented capacities for the primary fusion step since their respective myoblasts were able to fuse together as shown in Figure 2a,d. However, only WT RC were observed to fuse with nascent WT MT (Figure 2b), which is characteristic of the secondary fusion step. Moreover, the fusion of the primary MT, observed in WT (Figure 2c), also promoted the growth of mature MT, which constitutes properties of the secondary fusion step. In *Pofut1* knockdown context, after cell alignment, only an end to end multifusion of MB(d) was observed (Figure 2d) whereas Po – nascent MT were never shown to fuse together. These results suggest that knockdown of *Pofut1* induces secondary fusion and maturation defects.

### 2.2. Myonuclear Accretion Defect in Po – Cells is Independent of NOTCH Pathway Activation

**Figure 3:**
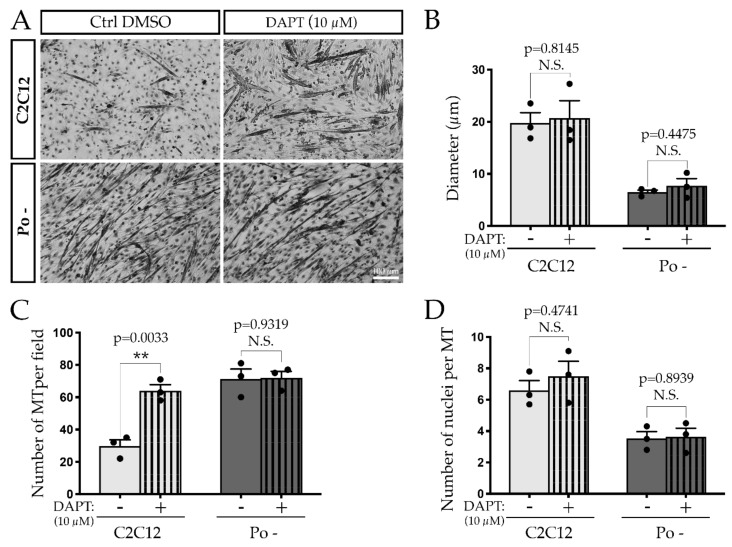
Secondary fusion does not rely on the NOTCH signaling pathway. Photographs show WT C2C12 and Po – cells at 120 h of differentiation treated with DMSO (the control – Ctrl) or DAPT at 10 μM (**A**). Although the inhibition of NOTCH signaling did not impact the diameter of the MT in any conditions (**B**) the number of MT per field increased in treated WT C2C12 cells and became similar to Po – ones (**C**). The number of nuclei per MT, reflecting the myonuclear accretion, was not modulated following the blockade of NOTCH intracellular domain (NICD) cleavage in both cell lines (**D**). Experiments were conducted in biological triplicates. Bar graphs represented means ± SEM. In B, C and D, each mean of replicates was symbolized by a dark dot. Statistical analyses were performed using a two-tailed Student test, which compared cell lines treated or not with DAPT. **, *p* < 0.01. N.S., not significant.

As POFUT1 impacts myogenic cell commitment through NOTCH signaling [22], alteration of this pathway could also be responsible of the secondary fusion defect observed in Po – cells. To challenge this hypothesis, the γ-secretase inhibitor DAPT (N-[N-(3,5-difluorophenacetyl)-l-alanyl]-(S)-phenylglycine t-butyl ester), which prevents proteolytic cleavage of NICD, was used at 10 μM during the first 120 h of WT C2C12 and Po – cell differentiation. This concentration was previously demonstrated to inhibit NOTCH signaling in WT C2C12 cells [22]. Phenotypic analyses (Figure 3A) revealed that at 120 h, WT C2C12 treated with DAPT present significantly more MT per field (64.0 ± 3.79) compared to C2C12 cells treated with an equivalent volume of DMSO (dimethyl sulfoxide), 29.7 ± 3.93 (Ctrl DMSO, Figure 3C). This increased number of MT in WT C2C12 following DAPT exposure is comparable to the number of Po – MT, which is unchanged by the treatment (Po –/DAPT: 72.0 ± 4.04; Po –/DMSO: 71.3 ± 6.12). However, neither the diameter (WT C2C12/DMSO: 19.78 ± 1.98 and WT C2C12/DAPT: 20.74 ± 3.32; Po –/DMSO: 6.50 ± 0.41 and Po –/DAPT: 7.72 ± 1.39, Figure 3B) nor the number of nuclei per MT (WT C2C12/DMSO: 6.6 ± 0.62 and WT C2C12/DAPT: 7.5 ± 0.95; Po –/DMSO: 3.5 ± 0.43 and Po –/DAPT: 3.6 ± 0.55, Figure 3D) were impacted following DAPT treatment on both C2C12 cell lines. Taken together, these results demonstrate that NOTCH is crucial for cell fate decision and determination of myoblasts primed for fusion, but not responsible for myonuclear accretion.

### 2.3. NFATc2/IL-4 Pathway Is Deregulated in Po—Cells

**Figure 4:**
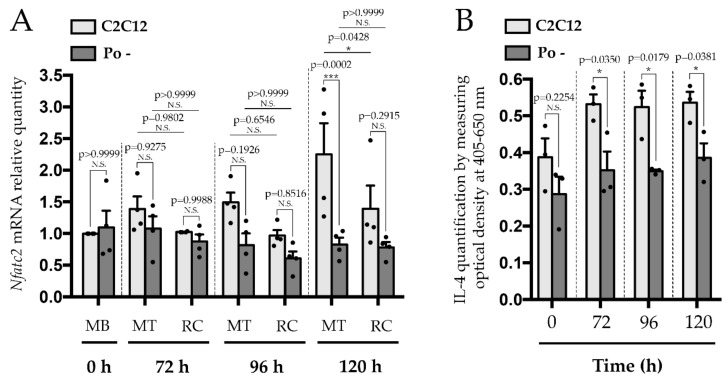
*Nfatc2* mRNA and IL-4 quantities are downregulated in Po – cells during secondary fusion process. qPCR analysis (**A**) indicates that *Nfatc2* was significantly decreased at 96 h and 120 h in Po – compared to WT C2C12 cells. Mouse IL-4 ELISA (**B**) performed on WT C2C12 and Po – cells during differentiation process shows that its presence in the culture medium was impaired in Po – cells. All experiments were carried out at least in biological triplicates and each mean of replicates was symbolized by a dark dot. Bar graphs represented means ± SEM. For both cell lines, fold changes were relative to time 0 h of WT C2C12. Statistical analyses were performed using a two-way ANOVA with p-value calculated by Sidak’s post-test (A) and a two-tailed Student test (B), which compared within differentiation times and according to both cell types, MT and RC. * *p* < 0.05; *** *p* < 0.001. N.S., not significant.

Numerous signaling molecules and pathways are involved in myogenic fusion processes, especially for the primary step [33]. However, those specifically involved in the secondary step of the fusion are not so abundant, with the exception of the NFATc2/IL-4 pathway. NFATc2 translocation into the nucleus directly controls IL-4 production. The presence of IL-4 in the myotubes’ secretome ensures the recruitment of RC during the secondary fusion process [10,12]. Lack of IL-4 or the IL-4Rα induces a peculiar MT phenotype close to the Po – one, with a reduced size and a lowest myonuclear number compared to WT [12]. We first determined the relative quantity of *Nfatc2* mRNA in WT C2C12 and Po – cells during the myoblast differentiation process. From 96 h, i.e., at the time of which the secondary fusion takes place, a decrease of *Nfatc2* mRNA quantity was observed in Po – compared to WT C2C12, especially in MT at 96 h (by 45%) and at 120 h (by 65%) (*p* < 0.001) (Figure 4A). Interestingly, at 120 h, a significant difference (*p* < 0.001) in *Nfatc2* expression is detected between MT and RC in WT C2C12, unlike Po –.

We also quantified the presence of IL-4 in the culture medium of WT C2C12 and Po – cells. A significant decrease (*p* < 0.05) in Po – compared to WT C2C12 was detected, especially from 72 h (around 30%) (Figure 4B), that corresponds to the notable apparition of MT in WT C2C12, up to 120 h, where the secondary fusion is still in progress (Figure 2b,c). In parallel to the altered *Nfatc2* profile of expression, Po – cells present an impaired IL-4 profile of expression, which could modify the expression of the IL-4 receptor.

**Figure 5:**
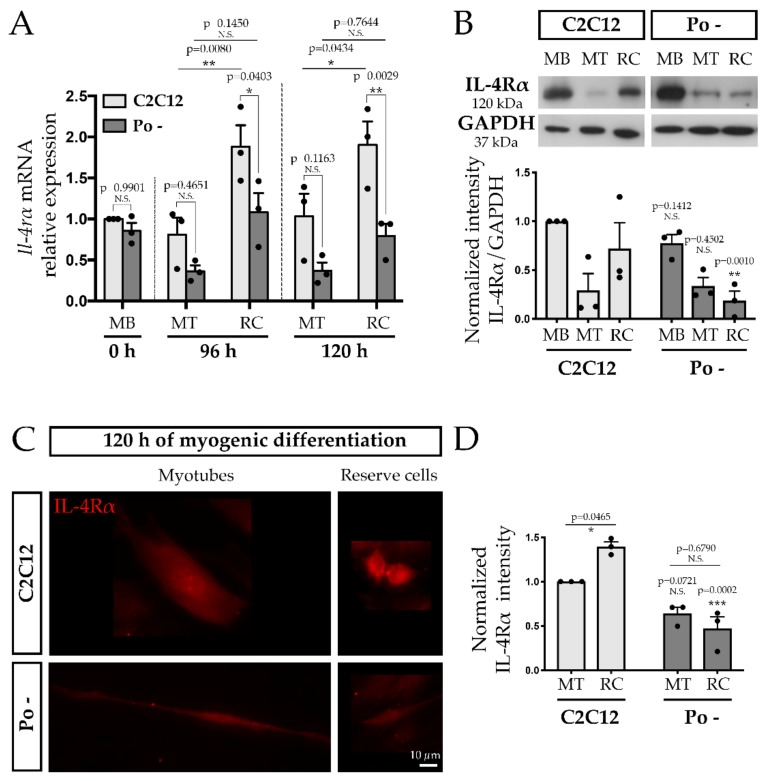
IL-4Rα expression for reserve cells decreases in Po – cells during differentiation time course compared to WT C2C12. qPCR analysis (**A**) performed on both cell lines highlighted that *Il-4rα* mRNA quantity is significantly decreased in Po – RC at 120 h, which is confirmed in terms of proteins by Western blot (WB) (**B**), immunofluorescence labelling (**C**) and its quantification (**D**). qPCR, WB and immunofluorescence experiments were done in biological triplicates and fold changes calculated relative to WT C2C12 cells, MB for qPCR and WB, and MT at 120 h for immunofluorescence quantification. Bar graphs represented means ± SEM. In (**A**), each mean of replicates was symbolized by a dark dot. Statistical analyses were performed using a two-way ANOVA with p-value calculated by Sidak’s post-, which compared within differentiation times and according to both cell types, MT and RC. * *p* < 0.05; ** *p* < 0.01; *** *p* < 0.001. N.S., not significant.

Thus, we followed the *Il-4rα* expression in WT C2C12 and Po – cells by qPCR during differentiation. We showed significant decreases by 42.4% at 96 h (*p* < 0.05) and 58.3% at 120 h (*p* < 0.01) in Po – RC compared to WT ones, while no significant difference was observed in MT (Figure 5A). For both cell lines, *Il-4rα* expression was higher in RC relative to MT, especially at 96 h where significant differences were observed (*p* < 0.01).

Western blot analysis performed on MB at 0 h, MT and RC at 120 h of differentiation only showed a significant drastic decrease (by 66.5%) of IL-4Rα in Po – RC compared to WT ones (Figure 5B). Furthermore, IL-4Rα immunofluorescence labeling confirmed its downexpression in Po – RC and a similar expression level in MT between WT C2C12 and Po – (Figure 5C,D). Taken together, these results establish that *Pofut1* knockdown cells present an impaired profile of *Nfatc2* expression profile and secrete a lesser amount of IL-4 than WT C2C12 during differentiation. It is correlated with a decrease of IL-4Rα expression in Po – RC, which would be unable to perform a correct secondary fusion with nascent myotubes.

### 2.4. Presence of IL-4 Receptor α at the Cell Membrane Is Necessary for the Recruitment and Fusion of Reserve Cells with Nascent Myotubes 

**Figure 6:**
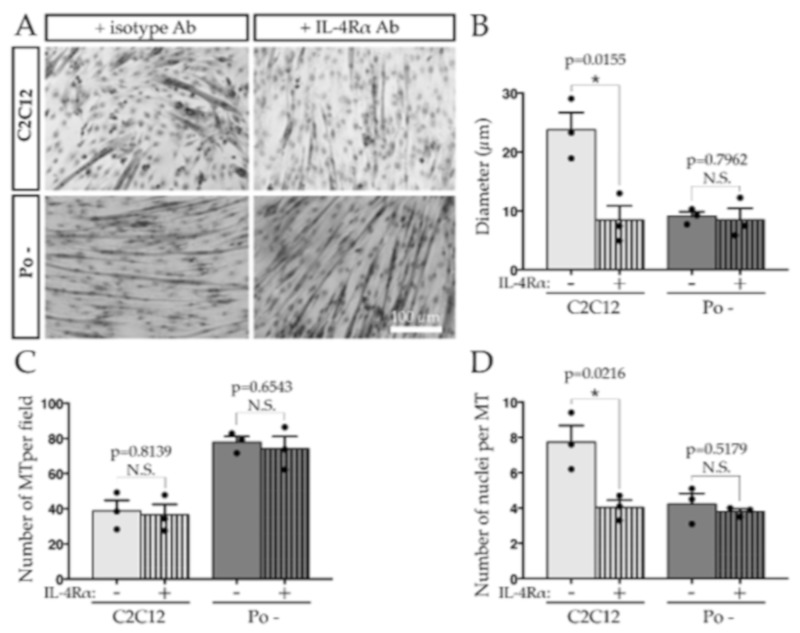
IL-4Rα is involved in the MT growth. Neutralizing assay with anti-IL-4Rα antibody on WT C2C12 cells generated thin and elongated MT like in Po –, observable at 120 h (**A**). WT C2C12 MT incubated with anti-IL-4Rα antibody during differentiation time course presented a lesser diameter than C2C12 untreated cells, similar to the diameter of Po – MT (**B**). Anti-IL-4Rα antibody treatment did not impact the number of MT per field in any cases (**C**) but in C2C12 treated cells, the number of nuclei per MT decreased and became similar to Po – one (**D**). Experiments were conducted in biological triplicates. Bar graphs represented means ± SEM. In **B**, **C** and **D**, each mean of replicates was symbolized by a dark dot. Statistical analyses were performed using a two-tailed Student test, which compared cell lines treated or not with anti-IL-4Rα antibody. * *p* < 0.05. N.S., not significant.

To determine if the defect of NFATc2/IL-4 pathway could be responsible of the Po – cell phenotype, neutralizing experiments were performed using anti-IL-4Rα antibodies or a rabbit IgG isotype control on WT C2C12 cells to block the recruitment of RC. Morphometric analysis and MT counting per field showed that at 120 h, WT C2C12 MT treated with anti-IL-4Rα antibody displayed a slender and elongated phenotype, closely resembling that of *Pofut1* knockdown MT (Figure 6A). Their diameter significantly decreased from 23.5 to 8.8 µm (Figure 6B). Conversely, the anti-IL-4Rα treatment of Po – cells did not affect the already elongated and slender phenotype of Po – MT nor their diameter (Figure 6A,B). The anti-IL-4Rα treatment did not impact the total number of MT counted per field in WT C2C12 (around 40) and Po – cells (around 80) (Figure 6C), but the number of nuclei per myotube was significantly lowered in treated WT C2C12, being comparable to the Po – one (around four, Figure 6D). It indicates that after the primary fusion process, the recruitment of RC involved at least the IL-4 secretion and its interaction with IL-4Rα to allow the growth of MT in WT C2C12.

## 3. Discussion

In this study, we revealed that the impaired expression of *Pofut1* gene encoding enzyme responsible for *O*-fucosylation of proteins, leads to defects of the secondary fusion process during myogenesis, in particular through NFATc2/IL-4 signaling pathway. Myogenic differentiation process is under the complex dependence of the transcriptional factors, MRFs (Myf5, MyoD, Myogenin and Myf6), whose expression is coordinated and highly specific for the skeletal muscle lineage. Myf6 is the later expressed MRFs, which makes it essential for organizing and maintaining myotubes triggering the expression of myotube-specific genes [34] and fundamental for the final phase of myogenic differentiation in postnatal myogenesis [1,35]. The significant difference in *Myf6* expression observed between WT C2C12 and the *Pofut1* knockdown cell line (Po –) at 120 h of the differentiation time course attests to a disturbed myotube maturation characteristic of an impaired secondary fusion in Po –. This result is confirmed by a reduced number of nuclei per myotube. Both WT C2C12 and Po – myoblasts are able to fuse together whereas peculiar nascent Po – MT were not able to fuse with surrounding Po – RC to form mature MT. So, POFUT1 is essential to the correct myoblast-myotube and myotube-myotube fusions. Myonuclear accretion is normally facilitated in the secondary fusion process by the recruitment of neighboring myoblasts, which express IL-4Rα at their cell surface. This recruitment is induced by nascent MT secreting interleukin 4 (IL-4) under the control of NFATc2 pathway [12]. *Pofut1* knockdown in C2C12 prevents the normal *Nfatc2* expression in MT and RC at the myogenic fusion time. It also impairs *Nfatc2* differential expression between the two cell types, necessary to the recruitment of RC by nascent MT. The secreted IL-4 is also decreased, which would notably affect the myonuclear accretion by Po – nascent MT. Conservation of an optimum IL-4 centripetal concentration gradient produced by nascent MT could be a key part to attract the surrounding RC. Moreover, IL-4 itself was involved in the expression of IL-4Rα receptor [14]. Its increased presence at the cell surface of WT RC could possibly involve STAT6, which can bind on the *Il-4rα* gene promoter and induce its expression [36]. IL-4 diminution in Po – would then explain the reduction of IL-4Rα for Po – RC. An interconnection was reported between NOTCH1 activation and calcineurin-NFAT pathways in keratinocyte differentiation control [32] and in T-cell acute leukemia malignancy and recidivism [37]. Furthermore, an exposure of osteoblasts to the NOTCH ligand DLL1 induces *Nfatc2* expression and this effect could be due to a post-transcriptional mechanism where NICD stabilized *Nfatc2* mRNA [31]. Although the secondary myogenic fusion is independent of NOTCH pathway activation, a link between *Nfatc2* expression and NOTCH signaling exist during proliferation (data not shown). Possibly, as in the osteoblasts, NICD, through the regulation of some miRNA expression, could contribute to stabilization of the *Nfatc2* mRNA, improving its half-life time [31]. The miRNA expression profile of mouse satellite cells was recently unraveled and highlighted several miRNA clusters, whose modulation of expression depends on lineage progression from quiescent to activated and differentiated satellite cells [38]. Among them, mmu-miR-329-3p and mmu-miR-190a-3p are highly expressed in quiescent satellite cells, presenting similarity with RC, but drastically downregulated in differentiated cells, such as MT. These miRNAs present strong evidence to target *Nfatc2* in 3’UTR region of murine embryonic stem cells [39] and hepatocytes [40], using a high-throughput sequencing of RNAs, isolated by cross-linking immunoprecipitation approach. To date, most studies have shown that miRNAs bind to a specific sequence at the 3′ UTR of their target mRNAs to induce translational repression and mRNA deadenylation and decapping. Indeed, the decrease of mmu-miR-329-3p and mmu-miR-190a-3p expression in differentiated muscle cells could explain the increase of *Nfatc2* expression in MT of WT C2C12 model. The protein *O*-fucosyltransferase 1, POFUT1, a soluble protein located in ER lumen [24,41], is responsible of *O*-fucose addition on S or T residue within the consensus sequence C^2^X_4_(S/T) C^3^ of EGF-like domain [25] of cell surface and secreted proteins [42]. A correct *O*-fucosylation of NOTCH receptors, which can include 17 *O*-linked fucoses on the 36 EGF-like of mouse NOTCH1 [43], is essential to their cleavage, i.e., NICD production. Otherwise, it is known that elevation in the intracellular calcium activates the phosphatase calcineurin, which in turn dephosphorylates NFATc2, allowing its nuclear translocation [44]. The intracytoplasmic calcium homeostasis is possibly impacted in Po – cell line because the POFUT1 overexpression was shown to disrupt the ER structure [22] and could result in modifying the intracellular calcium regulation. Moreover, many of the NOTCH EGF-like repeats bind calcium that imparts rigidity necessary to ligand interaction and successful receptor cleavage [45].

Among all proteins linked to myogenesis [46], several are implicated in myoblast polarization, like cadherins [47]. Among the few eighties POFUT1 predicted target proteins, CELSR1 to three (Cadherin EGF LAG seven pass G type receptors) proteins present up to eight EGF-like domains and two *O*-fucosylation potential sites per isoform, in human and mouse [48]. They belong to an atypical cadherin subfamily and play critical roles in neuronal cell differentiation, axon guidance, neuronal migration and cilium polarity [49]. Although CELSR are expressed in human skeletal muscle, they were poorly studied in the myogenesis context [50]. STAB2, another putative POFUT1 target containing in mouse 17 EGF-like domains with six potentially *O*-fucosylation sites, which contributes to cell-cell interactions [51,52], is implicated in myogenic fusion process. During myogenic differentiation, *Stab2*^-/-^ mouse myoblasts produce small and thin MT with a reduced number of nuclei per MT, highlighting the participation of STAB2 in myonuclear accretion [53]. This phenotype similar to that of Po – MT suggests that *Pofut1* knockdown could also impair STAB2 function in myogenic fusion. Thus, POFUT1 expression and *O*-fucosylation of its targets might be new controllers of the myogenic fusion process and should, as such, deserve more attention in research on skeletal muscular diseases.

Finally, the results exposed in this work were obtained from an *in vitro* model, the mouse myoblast C2C12 cell line. The next step would be to test if the NFATc2/Il-4 pathway is also affected in *in vivo* models. The knockout of *Pofut1* in mouse is lethal at midgestation with severe defects similar to those of mice in which canonical NOTCH signaling pathway is inactivated [26]. In 2009, a spontaneous mutation in *Pofut1* gene called *Pofut1*^cax^ was described in a mouse strain [54]. It corresponds to insertion of an intracisternal A particle (IAP) in the fourth intron of the *Pofut1* gene, leading to a hypomorphic allele and a decrease in gene expression without affecting protein structure and activity. Homozygous *Pofut1*^cax/cax^ mice are viable and display compact axial skeleton [54] consistent with a defect in Notch patterning during somitogenesis. Analysis performed on a culture of *Pofut1*^cax/cax^ myoblasts derived from satellite cells revealed a decrease in *Pax7* expression, a depletion of PAX7^+^/MYOD^−^ cells and a disruption of the myogenic process, leading to an earlier differentiation [23]. These observations were clearly similar to those of Po – cell line. However, a moderate hypertrophy was observed for skeletal muscles in *Pofut1*^cax/cax^ mice [23]. The fate of satellite cells was not analyzed following regeneration time course to evaluate the capacity of the oncoming migrating progenitors to fuse together or with nascent myotubes. As pre- and post- natal myogenesis involved distinct mechanisms, the *Pofut1*^cax/cax^ muscle hypertrophy could reveal the involvement of IGF/Akt/mTOR pathway [55] rather than NFATc2/IL-4 one. Thus, secondary fusion in myogenesis would be as the primary fusion a complex physiological process involving several key proteins as POFUT1 and dependent of various signaling pathways.

## 4. Materials and Methods

### 4.1. C2C12 Cell Lines and Culture

The murine myoblast C2C12 cell line was purchased from ATCC^®^ (American Type Culture Collection). *Pofut1* knockdown C2C12 cell line (Po –) was obtained as previously described [22]. Cells were cultured in a growth medium (GM) with Dulbecco’s modified Eagle’s medium (DMEM, Gibco® Life Technologies™, Carlsbad, CA, USA) supplemented with 10% fetal calf serum (Eurobio, Courtaboeuf, France), 50 units/mL penicillin, and 50 μg/mL streptomycin at 37 °C in 5% CO_2_. To induce differentiation of confluent cells, GM was removed and replaced by a differentiation medium (DM), which comprises DMEM supplemented with 2% horse serum, 50 units/mL penicillin, and 50 μg/mL streptomycin (Gibco® Life Technologies™). Samples named myoblasts (MB) correspond to cells at time 0 h, defined by replacement of GM with DM. Two cell types were collected from 72 h after DM addition. Indeed, a short trypsinization of 30 s in 0.1% trypsin, 0.1 mM EDTA allowed to remove myotubes (MT), the reserve cells (RC) being adherent to the flask. Then, a second trypsinization (0.1% trypsin, 0.1 mM EDTA; 2 min) allowed collecting them in turn, as previously reported [22].

### 4.2. Semi-Quantitative Real-Time Reverse Transcription-PCR

Total RNA was isolated using RNeasy mini Kit (Qiagen Inc., Hilden, Germany) and the reverse transcription was performed using high capacity cDNA Reverse Transcription Kit (Applied Biosystem™, Thermo Fisher Scientific, Waltham, MA, USA). Gene expression was determined by semi-quantitative real-time RT-PCR on QuantStudio 3 real-time PCR system (Applied Biosystem™) with Gene Expression Master Mix (Applied Biosystem™) using the following TaqMan™ probes: *Gapdh* Mm99999915_g1, *Il-4rα* Mm01275139_m1, *Myf6* Mm00435126_m1, *Nfatc2* Mm00477776_m1. The comparative threshold cycle (C_T_) method (ΔΔC_T_) was used to quantify mRNA [56] and the relative quantity was normalized with *Gapdh* reference gene. Statistical analyses were performed by comparison of each differentiation time relative to 0 h of WT C2C12 cells, which was set as 1.

### 4.3. Phenotypic Studies

Cells were fixed in Phosphate Buffer Saline (PBS) containing paraformaldehyde 4% (PFA) for 20 min and dried overnight in 70% ethanol. The number of MT, their diameter and the number of nuclei per MT were counted after hematoxylin/eosin staining in three independent experiments during a 120 h differentiation time course. For each morphometric study, six randomized visual fields at 100 X magnification were studied using ImageJ 1.45s software (Wayne Rasband, NIH, Bethesda, MD, USA) [57].

### 4.4. IL-4 Medium Concentration Measured by ELISA Test

Cells were seeded at 5000 cells/cm^2^ in six well plates in GM. After two days of proliferation, they were induced in differentiation with the DM. Then, the culture medium was collected and replaced every 24 h. After 120 h, IL-4 concentration in each collected medium was measured using the Murine IL-4 Mini ABTS ELISA Development Kit (PeproTech, Rocky Hill, NJ, USA) and following the manufacturer’s recommendations. Optical density measurement at 405 nm, corrected by a measure at 650 nm, that is directly related to the amount of IL-4 present in the sample was quantified via the FLUOstar Omega plate reader (BMG Labtech, Champigny sur Marne, France).

### 4.5. Neutralizing Antibody Assay

C2C12 cells were treated with 10 µg/mL of isotype control antibody (rabbit IgG, sc-2027, Santa Cruz Biotechnology, CA, USA) or rabbit anti-IL-4Rα antibody (S-20, sc-686, Santa Cruz Biotechnology) from 72 h to 120 h of myogenic differentiation. Then, the number of MT, their diameter and the number of nuclei per MT were determined at 120 h.

### 4.6. Western Blot 

Proteins were extracted from MB at 0 h, MT and RC populations at 120 h, of myogenic differentiation, in WT C2C12 and Po – cell lines using RIPA lysis buffer (50 mM Tris-HCl, 150 mM NaCl, 1% Triton X-100 (*v/v*), 0.5% sodium deoxycholate (*w/v*), 0.1% sodium dodecylsulfate (*v/v*), pH 8) and a cocktail of protease and phosphatase inhibitors (Roche Applied Science, Mannheim, Germany). Protein lysates were centrifuged at 12,000× *g* for 20 minutes at 4 °C and protein supernatant concentrations were determined using Pierce™ BCA protein assay kit (Thermo Scientific™, Rockford, IL, USA) with bovine serum albumin (BSA) as a standard. Equal amounts of proteins were resolved by SDS-PAGE and transferred onto a nitrocellulose membrane (GE Healthcare, Buckinghamshire, UK). Membranes were blocked with TBS (50 mM Tris, 150 mM NaCl, pH 7.6) supplemented with 0.1% Tween-20 (*v/v*) (TBST) and 5% (*w/v*) non-fat dry milk for 1 h at room temperature. They were probed with rabbit anti-IL-4Rα antibody (S-20) or with goat anti-GAPDH antibody (AF5718, R&D Systems, Minneapolis, MN, USA) diluted at 1:500 in TBST, 2.5% (w/v) non-fat dry milk overnight at 4 °C. After three washes with TBST, membranes were incubated with secondary antibodies (anti-rabbit or anti-goat HRP-conjugated IgG, Dako, Glostrup, Denmark) diluted at 1: 1000 in TBST, 2.5% (*w/v*) non-fat dry milk, for 1 h at room temperature. After three washes in TBST, reactive proteins were revealed by enhanced chemiluminescence using BM Chemiluminescence Western blotting substrate (peroxidase [POD]) (Roche Applied Science) and exposed to a film (Hyperfilm ECL, GE Healthcare).

### 4.7. Immunofluorescence Studies

At 120 h of differentiation, WT C2C12 and Po – cells were fixed with 4% PFA-PBS for 20 min and permeabilized with 0.1% Triton X-100–PBS for 30 min at 4 °C. To minimize nonspecific reactions, cells were saturated for 1 h at room temperature using PBS with 20% goat serum and washed three times with PBS. Immunolabeling was performed with the rabbit anti-IL-4Rα (S-20) or isotype (sc-2027) antibodies. Cells were rinsed and incubated 1 h at room temperature with Alexa Fluor® 594 conjugate polyclonal goat anti-rabbit IgG (Molecular Probes, Eugene, OR, USA) before mounting on slides. Photographs were taken using an epifluorescence microscope (Leica DMI4000B MM AF Imaging System) powered by MetaMorph (Universal Imaging Corp., Downingtown, PA, USA). The mean intensity of IL-4Rα staining for MT and RC were quantified using ImageJ [57].

### 4.8. Live Cell Imaging

Living cells were followed from 96 h to 120 h of myogenic differentiation by microscopy (Leica DMI4000B) powered by MetaMorph (Universal Imaging Corp.). WT C2C12 or Po – cells were photographed every 15 min by the Leica microscope using bright field mode to build the time-lapse.

### 4.9. Statistical Analysis

All the experiments were performed at least three times and the results were reported as the means ± standard error of the means (SEMs). Statistical comparisons were performed using two-tailed t tests or ANOVAs two-way with Sidak’s post-test (Figure 4A and Figure 5) implemented in GraphPad Prism 7 (GraphPad Software Inc., San Diego, CA, USA). A *p* value of 0.05 or less was statistically significant.

### 4.10. DAPT Cell Treatment

Wild-type C2C12 and Po – cells were induced to differentiate for 120 h in DM in the presence of dimethyl sulfoxide (DMSO) or 10 μM N-[N-(3,5-difluorophenacetyl)-l-alanyl]-(S)-phenylglycine t-butyl ester (DAPT) (Calbiochem, San Diego, CA, USA). Afterwards, phenotypic studies were performed as previously explained in Section 4.3.

## Supplementary Materials

Supplementary Materials can be found at https://www.mdpi.com/1422-0067/20/18/4396/s1.
